# Deep circumflex iliac artery flap maxillary reconstruction for juvenile trabecular ossifying fibroma: a case report and systematic review

**DOI:** 10.3389/froh.2025.1655774

**Published:** 2025-12-16

**Authors:** Jiang Huang, Peng-Li Chen, Wang-yong Zhu, Feng Zhang, Ping-An Wu, Yu-xiong Su

**Affiliations:** 1 Department of Dental Surgery, The University of Hong Kong-Shenzhen Hospital, Shenzhen, Guangdong, China; 2 Department of Stomatology, Shenzhen University General Hospital, Shenzhen University, Shenzhen, China; 3 Department of Surgery, Division of Otolaryngology, Head and Neck Surgery, The University of Hong Kong-Shenzhen Hospital, Shenzhen, China; 4 Division of Oral and Maxillofacial Surgery, Faculty of Dentistry, The University of Hong Kong, Hong Kong, China

**Keywords:** juvenile trabecular ossifying fibroma, virtual surgical planning, computer-assisted surgery, deep circumflex iliac artery flap, maxillary reconstruction

## Abstract

Juvenile trabecular ossifying fibroma (JTOF) is a rare benign fibro-osseous tumor predominantly affecting the maxillofacial region in children and adolescents. This study represented a case of maxillary reconstruction using deep circumflex iliac artery (DCIA) flap for JTOF and systematically reviewed the clinical characteristics and management of JTOF. A systematic review of published studies was performed. Information including clinical features, management, and follow-up was collected. We reported a 15-year-old male with maxillary JTOF. The patient underwent maxillectomy followed by DCIA flap reconstruction guided by 3D-printed surgical guides. No recurrence during 24-month follow-up period. The systematic review included 43 articles and 86 cases. Tumors involved the maxilla and mandible equally, with an average diameter of 4.8 cm. The overall recurrence rate was 23.9%, with a mean follow-up period of 39.2 months. We systematically reviewed the clinical features of JTOF and reported a case managed with DCIA flap maxillary reconstruction. Larger cohorts and extended follow-up are needed to determine the optimal management strategy for this rare disease.

## Introduction

Ossifying fibromas are benign fibro-osseous neoplasms affecting the jaws and craniofacial skeleton. They are classified into the conventional form (also called cemento-ossifying fibroma) and juvenile ossifying fibromas (JOF). According to the 2005 WHO classification of odontogenic tumors, JOF is further subdivided into juvenile psammomatoid ossifying fibroma (JPOF) and juvenile trabecular ossifying fibroma (JTOF) (1).

JTOF is characterized histologically by a cellular fibroblastic stroma containing immature trabeculae of woven bone. In contrast to JPOF, JTOF predominantly occurs in a slightly younger age group, with a predilection for the maxilla and mandible rather than the paranasal sinuses or orbital regions (2). Both JTOF and JPOF require surgical management. However, JPOF often exhibits more aggressive local behavior and higher recurrence rates, necessitating radical resection, whereas JTOF may respond better to conservative approaches (3, 4). While several systematic reviews on JPOF exist, comprehensive reviews on JTOF are scarce. Here, we presented a case of maxillary JTOF managed with deep circumflex iliac artery (DICA) flap reconstruction and provided a systematic review of the clinical characteristics, treatment and prognosis of JTOF.

## Methods

### Search strategy

A systematic literature search was conducted in the PubMed, Embase, Scopus, Ovid, and Cochrane databases for articles published from 2005 January to 2025 February. The search strategy was shown as follows: (juvenile ossifying fibroma) OR (juvenile trabecular ossifying fibroma) OR (trabecular ossifying fibroma) OR (trabecular variant ossifying fibroma) OR (trabecular juvenile ossifying fibroma).

Articles identified through reference list were also checked. If articles with languages other than English were selected, the authors were contacted for English translation.

### Selection of studies

Articles were initially screened based on the title and abstract. Then full texts were reviewed to confirm eligibility.

Inclusion criteria were as follows:

(1) Clinical trials, including randomized controlled trials, cohort studies, case-control studies, and cases reports.
(2) Publications reporting cases of JTOF.
(3) Publications providing sufficient clinical, radiological, histological information, and management of JTOF.

Exclusion criteria were as follows:

(1) Studies without English translation.

All studies were reviewed independently by two authors. In the event of disagreement between the two authors, a third author was introduced to review and the inconsistencies were resolved through discussion.

### Data extraction and analysis

Two authors independently extracted data covering the following information: patient sex and age, duration of the lesion prior to treatment, predominant location of the lesion, tumor size (largest diameter), clinical symptoms, coexistent pathology, radiological locularity appearance, radiodensity, radiological limits of the lesion, treatment performed, and follow-up. The extracted data were verified by a third author. Given the heterogeneity of the data, a meta-analysis could not be performed. All data were collected and assessed using descriptive statistics.

### Statistical analysis

Descriptive statistics were used to summarize demographic features, management, and follow-up outcomes of JTOF.

## Results

### Case report

A 15-year-old boy presented with left face swelling in the head and neck department. He complained of a progressive left facial swelling for two years and left nasal obstruction for one year, but denied diplopia, eye pain, or blurry vision.

**Figure 1:**
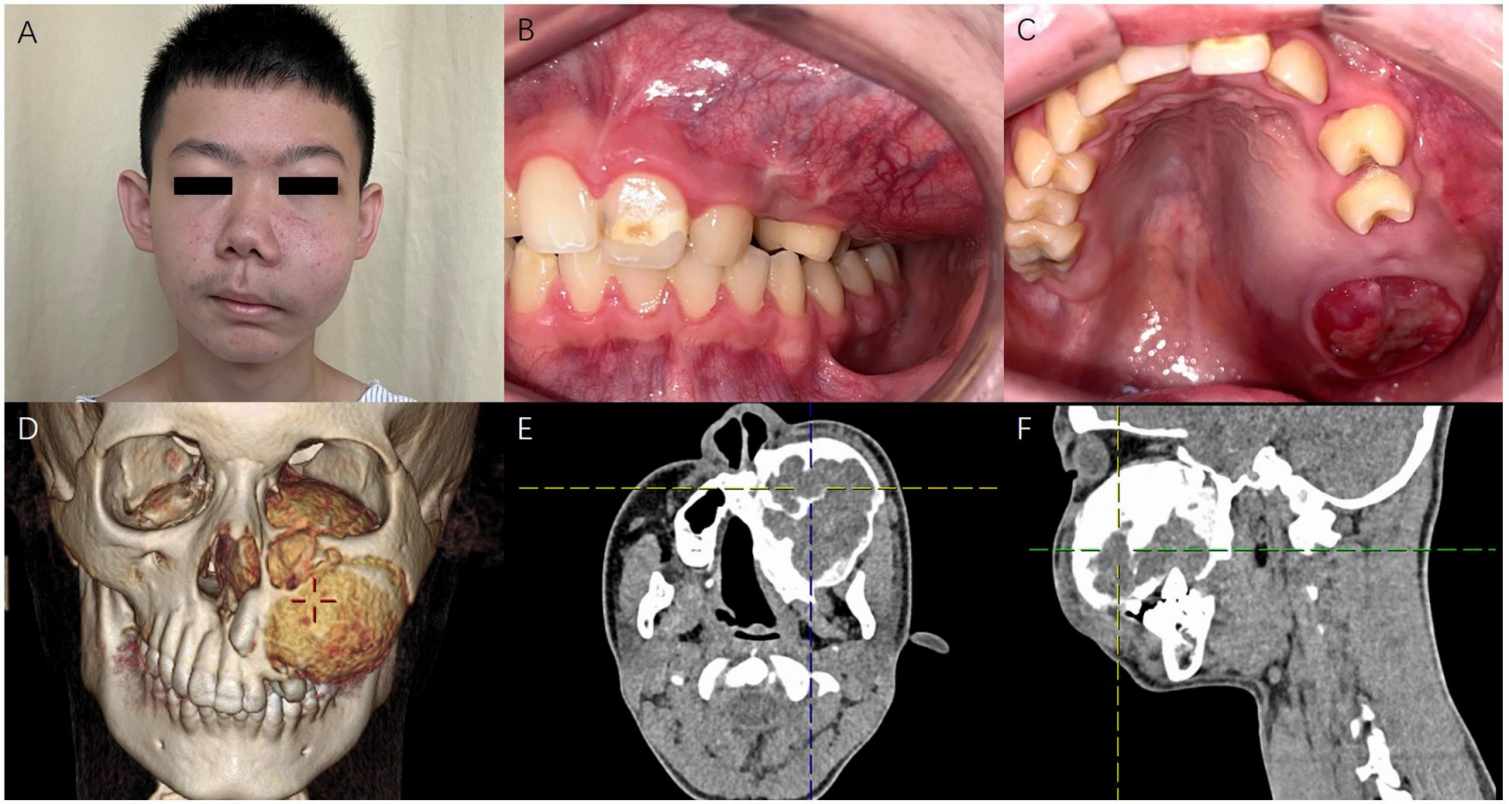
**(A)**, photograph of the patient displaying facial asymmetry due to left maxillary tumor. **(B,C)**, intraoral examination showing left maxillary mass. **(D–F)** maxillofacial CT revealed a 7.7 × 6.0 × 6.0 cm mass arising from the left maxilla, filling the left maxillary sinus. The mass was heterogeneous with mixed areas of density, with a clear and uneven thickness margin.

A physical examination revealed a firm, non-tender swelling left maxilla with mobile cheek skin. Intraoral examination revealed expansile left maxillary alveolar region, with missing #26, 27 and a bulging tumor instead. Nasal examination revealed a red, circular mass nearly occluding the left nasal cavity. Examination of the left eye showed mild proptosis and upward deviation. Maxillofacial CT with contrast revealed a 7.7 × 6.0 × 6.0 cm mass arising from the left maxilla and filling the left maxillary sinus. The mass was heterogeneous with mixed-density areas and exhibited a well-defined margin of uneven thickness. The mass had invaded the left maxillary sinus, ethmoidal sinus, sphenoidal sinus, left turbinates, and partial left zygoma. It also expended into left orbit, pushing the left eyeball upwards (Figure 1).

An incisional biopsy of the mass via an intraoral approach revealed a JOF. This case was discussed at a multidisciplinary treatment conference, where primary surgical excision with reconstruction was recommended.

Preoperative CT scans of the maxilla, fibula, and pelvis were obtained. The data were imported into Proplan CMF 3.0 software (Materialise, Leuven, Belgium) for 3D model reconstruction and virtual surgical planning. The chief surgeon determined the margins of bony resection considering the physical examination findings and preoperative imaging. To achieve good facial contour and provide sufficient space for dental implants, two-segment DCIA flap was chosen for maxillary reconstruction instead of three-segment fibula flap. The virtual surgical planning data was imported into the Materialise 3-Matic 13.0 (Materialise, Leuven, Belgium). The 3D model of right orbital bone was mirrored to reconstruct the left orbital floor. The patient-specific surgical guides were custom-designed based on the patient's maxilla and iliac crest models and then printed. All internal fixation titanium plates and titanium orbital floor mesh were pre-bent using 3D-printed models.

**Figure 2:**
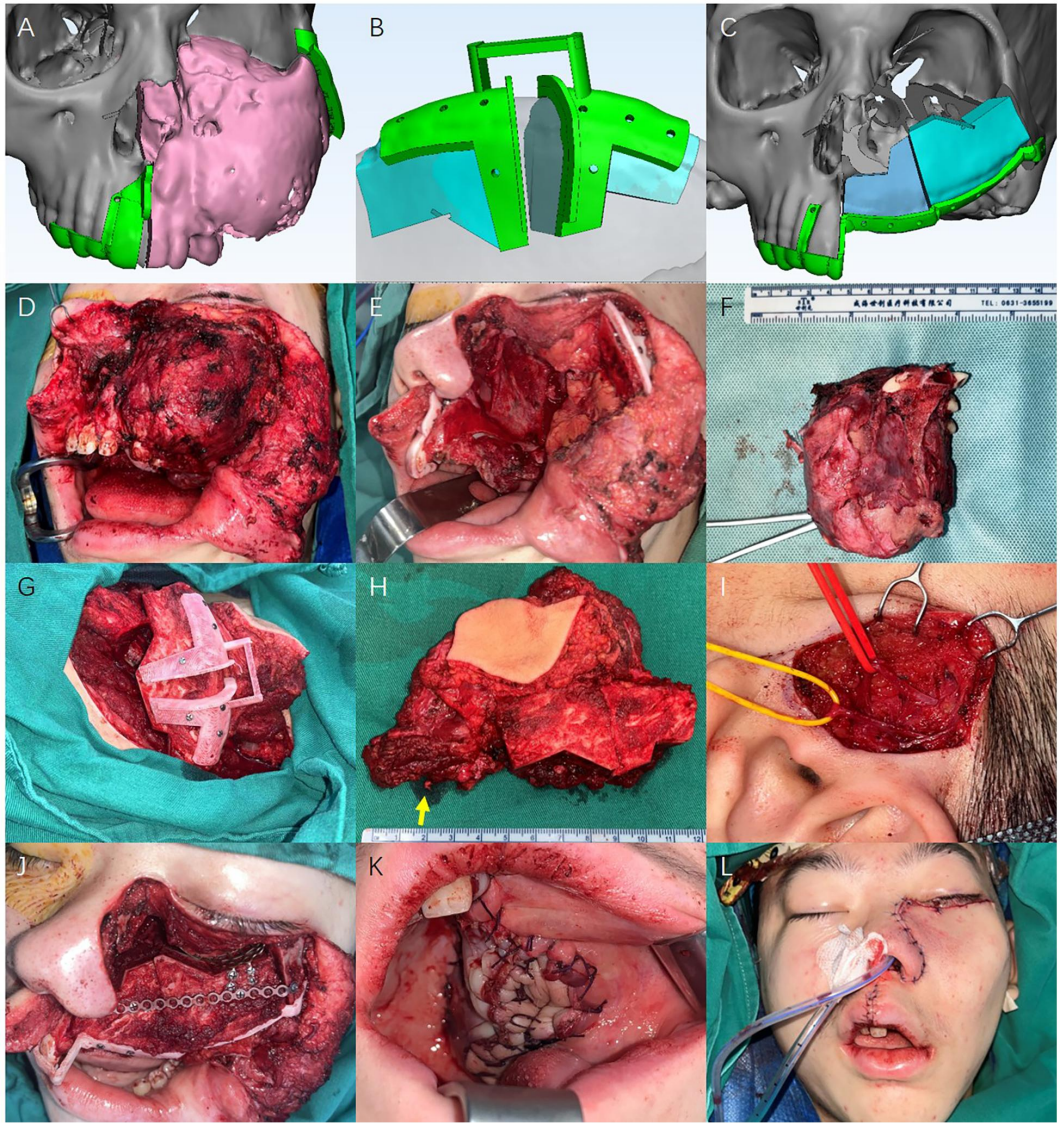
**(A–C)** The virtual surgical planning and design of patient-specific surgical guides on the patient's maxilla and iliac crest. **(D–F)** Tumor removal using patient-specific surgical guides. **(G)** The harvest of DCIA flap. **(H)** The harvested DCIA flap, with a bone size of 8.0 × 3.3 cm, a skin paddle size of 5.0 × 7.0 cm, and a vascular pedicle of 7.0 cm in length. (the arrow points the superior iliac deep arterial-venous vascular pedicle). **(I)** The superficial temporal artery and vein in the vascular recipient site. **(J)** The two-segments iliac bone was transferred to the recipient site and fixed, and the orbital floor was reconstructed using DCIA with pre-bent Titanium mesh. **(K)** The intraoral image of skin paddle. **(L)** Appearance of the patient after maxillary reconstruction.

The total operative time was 632 min, with a blood loss of 800 mL. We adopted a modified Weber-Ferguson incision including subsidiary incision for orbital exposure. Tumor was removed *en bloc* with total left maxillectomy. Intraoperative frozen section analysis confirmed clear surgical margins. The DCIA flap was harvested using a standard technique. The planned donor bone size was 8.0 × 3.3 cm, and the skin paddle size was 5.0 × 7.0 cm according to the maxillary defect, with a vascular pedicle measuring 7.0 cm in length. Osteotomies were performed according to the DCIA surgical guide. The iliac bone was then cut into two segments and fixed using the 3D printed temporal fixation guide. After division of pedicle, the flap was transferred to the recipient site and vessel pedicle was anastomosed to the left superficial temporal vessels. The orbital floor was reconstructed using DCIA with pre-bent Titanium mesh, which position was precisely designed according to the mirror image from contralateral side during virtual surgical planning. Intraoperative photos are shown in Figure 2.

**Figure 3:**
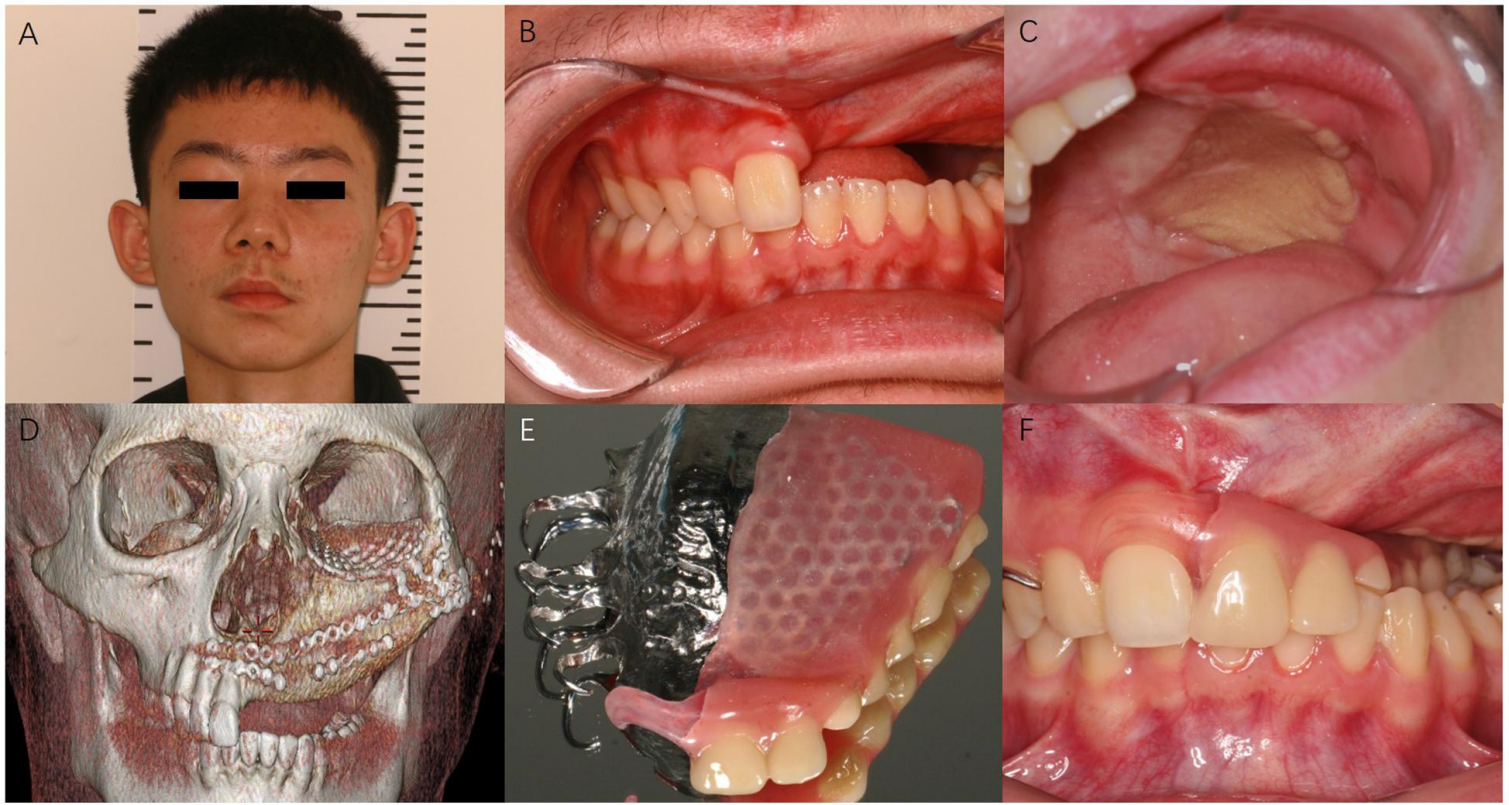
Postoperative 12 months follow-up. **(A)** Photography of the patient. **(B,C)** Intraoral examination showed maxillary flap. **(D)** Maxillofacial CT showed DCIA flap maxillary reconstruction and orbital floor reconstruction. **(E,F)** A removable partial denture was fabricated for the temporary oral function rehabilitation, before the permanent implant restoration.

Definitive histological analysis of all specimens confirmed a JTOF with complete resection. Postoperative recovery was uneventful, and the length of hospital stay was 17 days. Transient diplopia was noticed and recovered within one month. A removable partial denture was fabricated 9 months postoperatively. During the follow-up of 24 months, the patient's ocular function was well-preserved, without complications of diplopia, enophthalmos, or cicatricial ectropion. No reconstruction site or donor site complications occurred. The postoperative 12-month follow-up photo and radiographic images are shown in Figure 3.

### Results of the literature search

**Figure 4:**
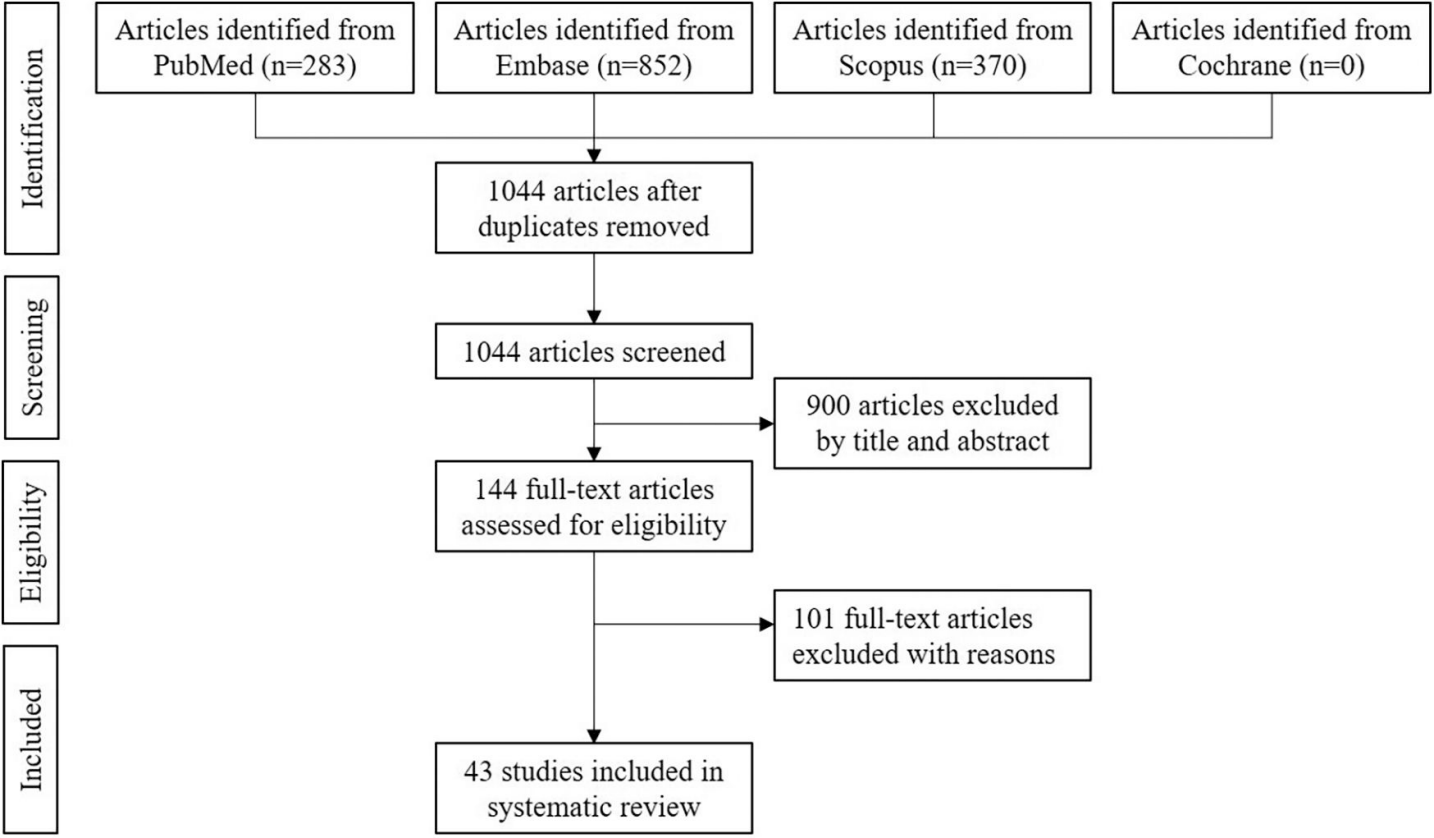
PRISMA flowchart of literature selection (From 2005 January to 2025 February).

283 articles were identified in PubMed, 852 in Embase, 370 in Scopus, and 0 in the Cochrane Library. After elimination of duplicated articles, 1,044 articles remained. 144 articles were selected based on the title and abstract, and were read in full text. Subsequently, 43 articles were included in systematic review (Figure 4) (5–47). All included articles were retrospective studies and case reports.

### Description of patient demographics

Demographic data from the included studies are presented in Table 1. A total of 86 patients were included. Among the cases with available data, 44 patients were male and 42 were female. The average patient age (available for 85 patients) was 12.1 years (range: 3–44 years). Thirty-eight studies with 62 patients reported the duration of the lesion prior to treatment. The averaged duration was 8.7 months (range: 0 month to 5 years). The predominant tumor location was reported for all 86 patients: 40 (46.5%) tumors located in the maxilla, 42 (48.8%) in the mandible, 3 (3.5%) in the nasal cavity, and 1 (1.2%) in the sellar region. Symptoms were reported in 70 patients, with swelling (88.6%) being the most common.

**Table 1:** Demographic characteristics of the included studies.

No.	Author & year	Sample size (n)	Sex (*n*)	Age (year)	Duration of the lesion (Month)	Location of tumor (*n*)	Largest diameter of tumor (cm)	Coexistent pathology (*n*)	Radiological locularity (*n*)	Radiodensity (*n*)	Radiological limits (*n*)	Treatment (*n*)	Surgical margin	Recurrence (*n*)	Time to recurrence (month)	Follow-up period (month)
1	G. Sun, 2007 (24)	1	M	11	0.5	Max	N.A.		N.A.	Mixed	Well-defined	Resection + reconstruction (orbital floor mesh)	N.A.	N.A.	N.A.	N.A.
2	P. R. Dominguete, 2008 (10)	1	F	18	12	Mand	N.A.		N.A.	N.A.	N.A.	1st: Resection, 2nd: Resection + reconstruction (iliac crest)	N.A.; Safe	Yes	36	72
3	M. Juneja, 2008 (15)	1	M	8	8	Max	10		N.A.	Mixed	N.A.	Resection	N.A.	N.A.	N.A.	N.A.
4	K. Banu, 2010 (28)	1	F	3	8	Mand	5		N.A.	Mixed	Well-defined	Excision	N.A.	No	N.A.	8
5	Y. Guruprasad, 2010 (13)	1	F	14	24	Max	5		Multilocular	Mixed	Well-defined	Peripheral ostectomy	Safe	No	N.A.	12
6	C. A. B. Silva, 2011 (44)	1	F	9	6	Max	N.A.	ABC	Multilocular	Radiolucent	Well-defined	1st: Excision, 2nd: Curettage	N.A.	Yes	36	42
7	R. Breheret, 2011 (8)	1	F	7	2	Max	4.2		N.A.	Mixed	Well-defined	Resection + reconstruction (orbital floor mesh)	N.A.	No	N.A.	9
8	S. M. B, N. Das, 2012	1	M	7	1	Mand	2		N.A.	Radiolucent	N.A.	Curettage	N.A.	N.A.	N.A.	N.A.
9	S. Rai, 2012 (40)	2	M 1; F 1	15 (15–15)	24, N.A.	Max 2	3		N.A.	Radiopaque 1; Mixed 1	Ill-defined 2	Curettage 1; Excision 1	N.A.	N.A.	N.A.	N.A.
10	P. J. Slootweg, 2012 (45)	15	M 6; F 9	13.9 (3–30)	N.A.	Max 9; Mand 6	N.A.		N.A.	N.A.	N.A.	Unknown surgery 15	N.A.	Yes 3; No 6; N.A. 6	23.3 (4–42)	110.7 (12–360); Recent 3; N.A. 3
11	M. C. Neidert, 2013 (34)	1	F	15	1	Sellar region	4		N.A.	N.A.	Ill-defined	Transsphenoidal and pterional endoscopic resection + radiotherapy (59.4 Gy)	Safe	No	N.A.	12
12	A. B. Urs, 2013 (47)	2	F 2	12 (10–14)	24 (12–36)	Max 1; Mand 1	4	ABC 2	Multilocular 2	Radiolucent 1; Mixed 1	Well-defined 1; Ill-defined 1	Excision 1; Resection 1	N.A.	No 2	N.A.	13.5 (12–15)
13	O. Osunde, 2013 (18)	1	F	7	9	Max	12		N.A.	Radiolucent	Well-defined	Resection	N.A.	N.A.	N.A.	N.A.
14	D. Gopinath, 2013 (12)	6	M 1; F 5	14.2 (11–19)	N.A.	Max 1; Mand 5	2–2.5	PaJOT 2	N.A.	Radiolucent 3; Mixed 3	N.A.	Excision 6	N.A.	No 4; N.A. 2	N.A.	50 (48–54); N.A. 2
15	O. A. Marglani, 2014 (31)	1	F	11	3	Nasal cavity	6		N.A.	Radiolucent	Ill-defined	Transnasal endoscopic resection	N.A.	No	N.A.	9
16	N. Pandit, 2014 (37)	1	M	13	2	Mand	N.A.		N.A.	Radiolucent	Ill-defined	Excision + curettage	N.A.	No	N.A.	3
17	A. V. Reddy, 2014 (20)	1	F	16	4	Mand	5		Multilocular	Radiolucent	Well-defined	Resection	N.A.	No	N.A.	24
18	S. H. Srivathsa, 2014 (23)	1	M	5	12	Mand	2		Multilocular	Radiolucent	Well-defined	Excision	N.A.	No	N.A.	6
19	T. Rahman, 2015 (39)	1	M	8	1	Max	5		N.A.	N.A.	Well-defined	Declined	N.A.	N.A.	N.A.	N.A.
20	S. Aboujaoude, 2016 (26)	1	F	7	N.A.	Max	2.5		N.A.	Mixed	Well-defined	Excision	N.A.	No	N.A.	24
21	J. Han, 2016 (14)	10	M 4; F 6	12.4 (7–20)	18 (0–60)	Max 4; Mand 6	2–10	ABC 2	Unilocular 1; Multilocular 2; N.A. 7	Radiolucent 3; Radiopaque 1; Mixed 4; N.A. 2	Well-defined 7; N.A. 3	Curettage 3; Resection 1; Curettage/resection + reconstruction (fibula) 2; Curettage/resection + reconstruction (iliac crest) 3; Resection + reconstruction (costal cartilage) 1	N.A.	Yes 4; No 6	7.8 (6–11)	63.4 (15–146)
22	L. Bhuyan, 2017 (7)	1	F	20	1.5	Max	5	PaJOT	N.A.	Mixed	Ill-defined	Excision	Safe	No	N.A.	6
23	F. Fauvel, 2017 (11)	1	M	5	1	Mand	6		Multilocular	Radiolucent	Well-defined	Resection + reconstruction (costal cartilage)	N.A.	No	N.A.	12
24	P. Malaviya, 2017 (17)	1	F	17	2	Mand	6		Unilocular	Mixed	Well-defined	Resection + reconstruction (reconstruction plate)	N.A.	N.A.	N.A.	N.A.
25	A. Paranthaman, 2017 (19)	1	F	13	4	Max	3		N.A.	Mixed	Well-defined	Resection + skin grafting	N.A.	N.A.	N.A.	N.A.
26	S. Seifi, 2018 (43)	1	M	7	12	Mand	4		N.A.	Mixed	Ill-defined	Enucleation + curettage	N.A.	No	N.A.	6
27	A. S. Sultan, 2018 (46)	1	M	8	12	Mand	N.A.		Unilocular	Radiolucent	Well-defined	1st: Excision, 2nd: Enucleation + osteotomy	N.A.	Yes	6	6
28	N. K. Chaurasia, 2018 (9)	1	M	7	3	Max	3		Unilocular	Radiolucent	Well-defined	Debridement	N.A.	No	N.A.	3
29	J. Khanna, 2018 (16)	1	M	8	6	Mand	7		N.A.	Mixed	N.A.	Resection + reconstruction (reconstruction plate)	Safe	No	N.A.	36
30	H. Rizk Saad, 2019 (21)	1	F	9	12	Mand	N.A.	CGCG	Multilocular	Mixed	Well-defined	Excision	N.A.	No	N.A.	1.5
31	H. M. M. Sadeghi, 2021 (41)	1	M	13	1	Max	2		N.A.	Radiolucent	Ill-defined	Enucleation	N.A.	No	N.A.	14
32	F. Titinchi, 2021 (25)	10	M 7; F 3	10.1 (3–19)	1.4	Max 3; Mand 6; Nasal cavity 1	6.5		Unilocular 7; Multilocular 3	Radiolucent 2; Radiopaque 2; Mixed 6	Well-defined 8; Ill-defined 2	Enucleation 1; Curettage + peripheral ostectomy 5; Resection 4	N.A.	Yes 2;No 8	20.5	21.2
33	N. G. Nikitakis, 2022 (35)	5	M 3; F 2	11.8 (6–16)	2.6 (2–3)	Max 3; Mand 2	2–8		N.A.	Mixed 5	Well-defined 5	Enucleation 3; 1st: Enucleation 2nd: Resection; 1st: Enucleation 2nd: Excision + peripheral ostectomy	N.A.	Yes 2; No 3	6.5 (6–7)	10 (10–10)
34	K. A. Nnko, 2022 (36)	1	F	8	36	Max	12		Unilocular	Radiolucent	Well-defined	Resection	N.A.	No	N.A.	36
35	M. Nedelec, 2023 (33)	1	M	14	N.A.	Max	N.A.		N.A.	Mixed	N.A.	Curettage + secondary reconstruction (iliac crest)	Safe	No	N.A.	60
36	B. Sivapathasundharam, 2023 (22)	1	M	44	2	Max	N.A.	PaJOT	N.A.	N.A.	N.A.	Excision	N.A.	N.A.	N.A.	N.A.
37	K. A. Bishen, 2024 (29)	1	F	14	2	Mand	3	CGCG	N.A.	Radiolucent	Ill-defined	Excision	N.A.	N.A.	N.A.	N.A.
38	R. Krishna, 2024 (30)	1	M	14	22	Mand	8	CGCG and ABC	Multilocular	Mixed	Well-defined	Resection + reconstruction (reconstruction plate)	Safe	No	N.A.	12
39	D. A. A. Marlière, 2024 (32)	1	M	13	24	Mand	N.A.		N.A.	Mixed	Well-defined	1st: Enucleation + curettage + peripheral osteotomy, 2nd: Curettage + peripheral osteotomy	N.A.	Yes	12	24
40	D. Prabhu Venkatesh, 2024 (38)	1	F	7	1	Max	N.A.		N.A.	Radiolucent	Well-defined	Excision + curettage	N.A.	No	N.A.	16
41	M. Sagar, 2024 (42)	1	M	Early adolescent	12	Nasal cavity	3.3		N.A.	Radiopaque	Ill-defined	Transnasal endoscopic resection	N.A.	No	N.A.	3
42	S. Anjana, 2024 (5)	1	M	13	2	Mand	7		N.A.	Mixed	Ill-defined	1st: Curettage, 2nd: Resection + reconstruction (unknown)	N.A.	Yes	36	36
43	C. H. Bercu, 2024 (6)	1	F	5	12	Max	7.5		N.A.	N.A.	N.A.	Resection + reconstruction (calvarial bone)	Safe	No	N.A.	18

ABC, aneurysmal bone cyst; CGCG, central giant cell granuloma; F, female; M, male; Mand, mandible; Max, maxilla; N.A., not available; PaJOT, juvenile psammomatoid ossifying fibroma.

The largest diameter was reported for 46 tumors in 31 studies, with an averaged diameter of 4.8 cm, (range: 1–12 cm). Radiological examination revealed a mixed radiolucent and radiopaque appearance in 56.3% (36/64) of cases, a radiolucent appearance in 35.9% (23/64), and a radiopaque appearance in 7.8% (5/64). Most cases (41/55) presented a well-defined radiological margin. Hybrid pathology was reported in 9 studies: six cases reported coexistent aneurysmal bone cyst (ABC), four cases reported coexistent JPOF, and three cases reported coexistent central giant cell granuloma (CGCG).

### Managements and outcomes

Management was reported for all cases. Eighty-five cases underwent surgical treatment, except one case declined treatment. Sixteen cases underwent resection with reconstruction, including autogenous bone reconstruction (vascularized fibular flap in 2 cases, non-vascular iliac crest bone graft in 5 cases, non-vascular costal cartilage bone graft in 2 cases, and non-vascular calvarial bone graft in 1 case), the use of reconstructive implants (reconstruction plates in 3 cases, and orbital floor meshes in 2 cases), and unknown approach (1 case). Three cases underwent endoscopic resection, one of which received adjuvant radiotherapy.

Follow-up data was available for 67 cases, with a mean follow-up period of 39.2 months (range: 1.5–360 months). The recurrence rate was 23.9% (16/67), with a mean time to recurrence of 17.6 months (range: 4–42 months). Surgical margin was reported for only 8 cases, with no recurrence recorded. None of cases underwent resection with reconstruction reported recurrence (0/13), with a mean follow-up period of 44.8 months (range: 9–146 months).

## Discussion

In this report, we presented a young patient with maxillary JTOF reconstructed using a DCIA flap guided by 3D-printed patient-specific devices, followed by oral rehabilitation. Additionally, we provided a systematic review of the clinical features and management of JTOF.

Although termed “juvenile”, JTOF predominantly affects children and adolescents with a mean age of 12.1 years, but cases have been reported across a broader age range, including up to 44 years (22, 48). It usually presents as a painless, rapidly expanding mass, primarily in the maxilla and mandible. Bone expansion without cortical perforation is common, and tooth displacement occurs more frequently than root resorption (4, 25).

Histologically, JTOF exhibits hypercellular fibroblastic stroma containing immature, curvilinear trabeculae of woven bone lacking osteoblastic rimming. The stroma may contain osteoclast-like giant cells. Secondary ABCs are observed in approximately 7% of cases, and hybrid lesions of CGCG and JTOF are rarely reported in around 3% of cases (30, 49).

JTOF lesions presented high rates of recurrence (40%–62.5%) after treatment by curettage and enucleation only. Radical resection achieved the lowest recurrence rate (0%–10%) but risked significant morbidity. Enucleation followed by peripheral osteotomy/curettage demonstrated an acceptable recurrence rate (10%–33.3%), and was considered a less invasive treatment option to avoid the disfigurement often associated with extensive surgical resection (3, 4, 25). Half of the cases (16/30) with resection also received reconstruction, including fibula flap, iliac crest flap, orbital floor mesh, and reconstruction plate, to restore the function and appearance. In our review, recurrence typically occurred from 4 to 42 months postoperatively. Long-term follow-up is crucial, particularly for lesions with incomplete excision. M. C. Neidert et al. reported a transsphenoidal and pterional endoscopic resection for sellar JTOF, followed by radiotherapy, and no recurrence at 12 months follow-up (34).

The main limitation of the systematic review was the heterogeneity of included studies. Variability in reported clinical features, radiological characteristics, managements, and outcomes limited the ability to pool data and draw unified conclusions. Another limitation of the case report was the relatively short follow-up period. At 24 months of follow-up, the patient was not yet an adult, and his development of maxilla and mandible has not finished yet. The long-term outcome, the adaptation of the DCIA flap in the developing maxilla, and secondary placement of dental implants, require longer observation time to draw reliable conclusions.

## Conclusions

We systematically reviewed the clinical characteristics and managements of JTOF, and presented our initial experience using 3D-printed patient-specific devices for DCIA flap maxillary reconstruction and subsequent oral rehabilitation in a young patient with JTOF. Further studies with larger sample sizes are needed to understand the optimal management strategy and long-term outcomes for this rare disease.

## Data Availability

The raw data supporting the conclusions of this article will be made available by the authors, without undue reservation.
